# Breakthrough pain among cancer patients at oncology units in Northern Ethiopia; a multi-center study

**DOI:** 10.3389/fonc.2023.1248921

**Published:** 2024-01-09

**Authors:** Yohanes Tekie, Yonas Addisu Nigatu, Wudie Mekonnen, Yophtahe Woldegerima Berhe

**Affiliations:** ^1^ Department of Anesthesia, Aksum University, Aksum, Ethiopia; ^2^ Department of Anesthesia, University of Gondar, Gondar, Ethiopia

**Keywords:** breakthrough pain, cancer pain, cancer, pain, breakthrough cancer pain (BTCP)

## Abstract

**Background:**

Breakthrough cancer pain (BTCP) is a transient exacerbation of pain that affects the length of hospitalization and quality of life of patients. The objective of this study was to determine the prevalence and factors associated with BTCP among cancer patients at oncology units in Northern Ethiopia in 2022.

**Methods:**

A multi-center cross-sectional study was conducted from April to June 2022. After obtaining ethical approval, data were collected prospectively from 424 adult cancer patients admitted to oncology units. Breakthrough cancer pain was assessed by the numeric rating scale. Descriptive and binary logistic regression analyses were performed to determine the factors associated with BTCP. The strength of association was described in adjusted odds ratio (AOR) with 95% confidence intervals and variables with a P-value < 0.05 were considered to have a statistically significant association with BTCP.

**Result:**

The prevalence of BTCP among cancer patients was 41.5%. The factors that were found to be associated with BTCP were colorectal cancer (AOR: 7.7, 95% CI: 1.8, 32.3), lung cancer (AOR: 6.9, 95% CI: 1.9, 26.0), metastasis (AOR: 9.3, 95% CI: 3.0, 29.1), mild background pain (AOR: 7.5, 95% CI: 2.5, 22.6), moderate background pain (AOR: 7.0, 95% CI: 2.2, 23.1), severe background pain (AOR: 7.1, 95% CI: 2.2, 22.8), no analgesics taken for background pain (AOR: 5.1, 95% CI: 2.8, 9.3) and uncontrolled background pain (AOR: 3.3, 95% CI: 1.8, 6.1).

**Conclusion:**

The prevalence of BTCP was high. Colorectal cancer, lung cancer, the presence of metastasis, the presence of background pain, not taking analgesics for background pain, and uncontrolled background pain were significantly associated with BTCP.

## Introduction

Cancer is a multi-symptomatic disease associated with physical, social, and emotional sequelae, and it is one of the leading causes of death worldwide (1). Overall, nearly 10 million deaths were reported in 2020. Data from the World Health Organization’s Global Cancer Observatory (GLOBOCAN) showed that breast (2.26 million), lung (2.21 million), and colorectal (1.93 million) cancers were the most frequent new cases in 2020 (2). In sub-Saharan Africa in 2020, breast and cervical cancer were the most common, and prostate cancer was the leading incidence of cancer in men (3).

Cancer pain is one of the most complex symptoms due to its cognitive, behavioral, sensory, and emotional components. It is the most frightening symptom with a prevalence of 50% to 70%. The highest prevalence of pain is commonly observed among pancreatic and head/neck cancer (4–7).

Breakthrough cancer pain (BTCP) is defined as a transient exacerbation of pain that occurs either spontaneously or in relation to a specific, predictable, or unpredictable trigger, despite having relatively stable and adequately controlled background pain (8). It is a common clinical condition in cancer patients characterized by rapid onset, short duration, and severe intensity. It has a significant impact on a patient’s quality of life (9–11). Breakthrough cancer pain is categorized as spontaneous pain and incidental pain. Spontaneous pain is unpredictable and its precipitants are not clearly identifiable, whereas incidental pain is somewhat predictable (12–14). The consequences of poor breakthrough cancer pain management include poor functional ability, poor sleep patterns, increased anxiety and depression, poor compliance with cancer treatments, increased costs of care, and patient dissatisfaction (9, 15–17). A variety of factors such as sociodemographic factors, the type and site of cancer, stage, metastasis, physical activity, background pain, and pain medications have been found associated with BTCP (4, 18–29).

Breakthrough cancer pain is commonly overlooked by healthcare providers. Despite its deleterious consequences, the management of BTCP is still sub-optimal (5). Limited research-based evidence is available regarding BTCP in low- and middle-income countries, particularly in sub-Saharan Africa. The general objective of this study was to assess the prevalence and factors associated with BTCP at oncologic units in Northern Ethiopia.

## Methods

### Study design, period, and area

A multi-center cross-sectional study was conducted from 1 April to 28 June 2022. Three oncology units are based in Northern Ethiopia, including the University of Gondar Comprehensive Specialized Hospital (UoGCSH), Felege Hiwot Comprehensive Specialized Hospital (FHCSH), and Dessie Comprehensive Specialized Hospital (DCSH).

### Study populations, and inclusion and exclusion criteria

All adult (18+) cancer patients admitted to oncology units in the aforementioned hospitals in the Amhara National Regional State during the study period were included. Patients who were unable to communicate and volunteer to participate in the study were excluded.

### Variables and operational definitions

The dependent variable was breakthrough cancer pain and the independent variables were socio-demographic characteristics (age, sex), cancer characteristics (type, site, stage, and metastases), treatment-related factors, and background pain intensity.

**Breakthrough pain:** a transitory increase in pain to a score greater than NRS ≥ 7/10, which occurred alongside or further to a background pain (30).

**Background pain:** a mild to moderate intensity of the pain with a value of 1 to 6 on NRS (31).

**Controlled background pain:** background pain that is managed with scheduled opioids and non-opioid analgesics and has an NRS of < 3/10 (32).

**Numerical Rating Scale:** A pain assessment tool in which a number is assigned from 0 – 10 to represent the severity of the pain; 0 = no pain; 1 – 3 = mild pain 4 – 6 = moderate pain 7 – 10 = severe pain (33).

**Somatic pain:** Pain arising from the skin, subcutaneous tissue, and musculoskeletal system characterized by being intense, constant, and localized to a particular area (34).

**Visceral pain**: Pain originating from internal organs characterized by a diffuse, dull, and poorly localized type of pain (35).

**Neuropathic pain**: Pain resulting from damage to the nervous system characterized by burning, electrical shocks, shooting, and needle-type pain (36, 37).

### Sample size determination and sampling technique

**Figure 1:**
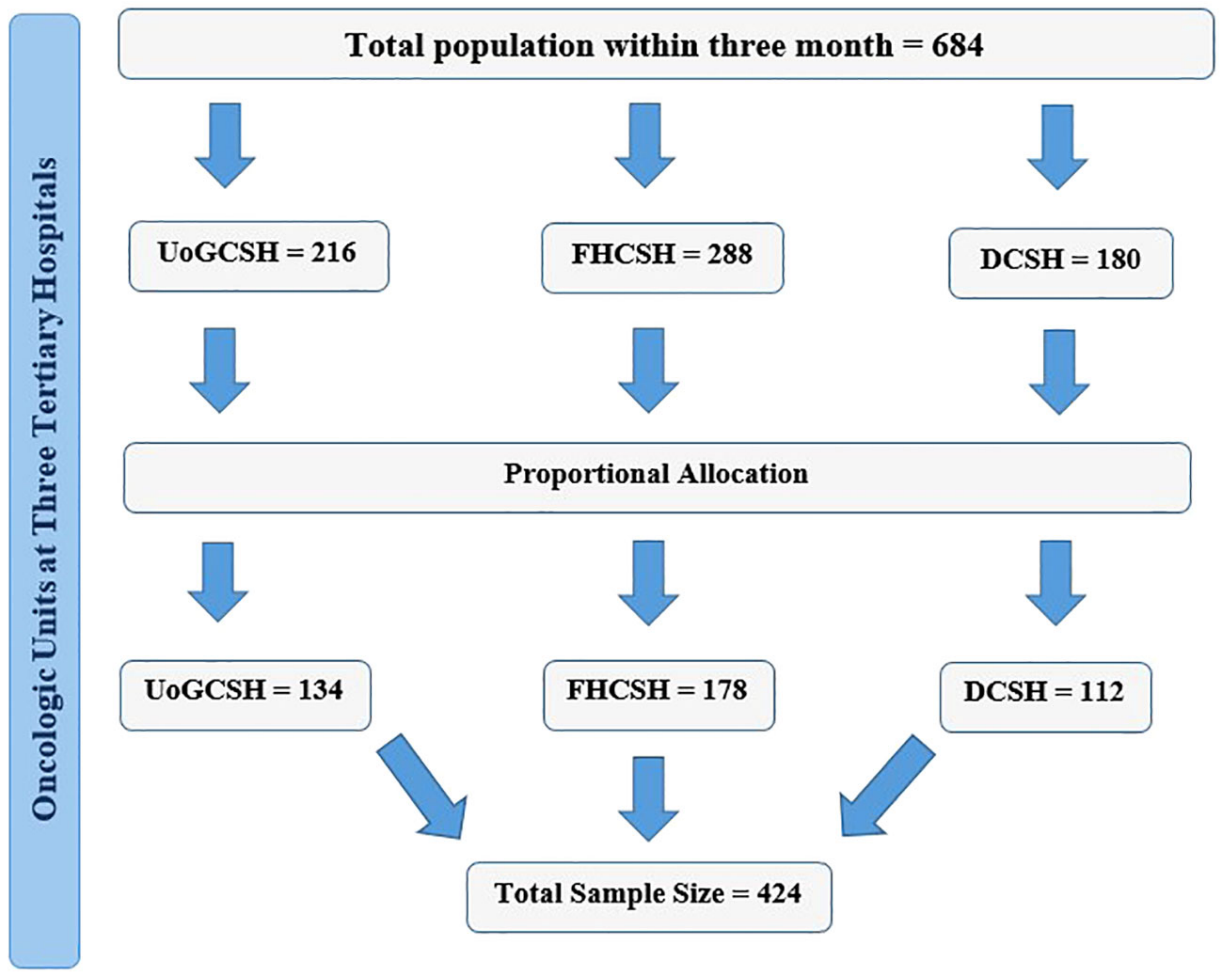
Sample selection procedure.

The sample size was determined by using a single population proportion formula with the assumptions of 50% prevalence rate and 5% margin of error at 95% confidence level. The final sample size was 424 with the addition of 10% non-response rates. All consecutive cancer patients who were admitted to those oncology units were included in this study center until the calculated sample size was attained. There were 2,736 cancer patients receiving cancer treatment follow-up in the three oncology units (864 at UoGCSH, 720 at FHCSH, and 1,152 at DCSH). The sample taken from each unit was calculated by using a proportion formula (134 UoGCSH, 112 FHCSH, and 178 from DCSH) (**Figure 1**). 

### Ethical approval, data collection procedure, quality control, and analysis

Ethical approval was obtained from the Ethical Review Committee of the School of Medicine, University of Gondar (**Reference number: SOM/1404/2022**), and permission to conduct data collection was obtained from each hospital. Written informed consent was obtained from each study participant after a detailed explanation of the study. All methods were performed in accordance with the relevant research guidelines and regulations.

The data were collected by three trained nurses by interviewing the participants and chart review, using a structured questionnaire. The questionnaire was adapted from the Alberta Breakthrough Pain Assessment. It was found valid and reliable to assess BTCP (Cronbach’s alpha = 0.83) (19, 32, 38, 39). To ensure the quality of the data, a pretest was conducted on 22 patients (5% of the sample). The accuracy, completeness, and consistency of the collected data were checked; and data collectors were closely supervised by the investigators.

The collected data were cleaned, coded, entered, and analyzed by SPSS version 26 (IBM Corporate). The normality of the data were tested using the Shapiro-Wilk normality test. Variables with variance inflation factors of less than 10 were considered to have no collinearity. The fitness of the model was checked using the Hosmer-Lemeshow goodness-of-fittest. Descriptive, Chi-square, and binary logistic regression analyses were employed to determine the factors associated with BTCP. Variables with a P value < 0.2 on bivariate logistic regression were entered into multivariate logistic regression analysis. In the multivariable logistic regression analysis, variables with a p-value of < 0.05 were considered to have a statistically significant association with BTCP. The strength of association was described in adjusted odds ratio (AOR) at a 95% confidence interval (CI).

## Results

### Socio-demographic and clinical characteristics

A total of 424 patients were incorporated into this study (100% response rate), with 265 (62.5%) female patients. The median age was 50.0 years (interquartile range = 39 – 62 years). Background pain was present among 380 (89.6%) patients, out of whom 121 (28.5%) were experiencing severe pain. Of the total, 259 (61.1%) patients took analgesics for background cancer pain (**Table 1**).

**Table 1:** Socio-demographic and clinical characteristics, and cross-tabulations with breakthrough cancer pain, N = 424.

Variables	Categories	Frequency n (%)	Breakthrough cancer pain	
			Yes n (%)	No n (%)
Age	18 – 64	322 (75.9)	135 (41.9)	187 (58.1)
	≥ 65	102 (24.1)	41 (40.2)	61 (59.8)
Sex	Male	159 (37.5)	72 (45.3)	87 (54.7)
	Female	265 (62.5)	104 (39.2)	161 (60.8)
Stage of the cancer	Stage I	133(31.4)	104(78.2)	29(21.8)
	Stage II	162(38.2)	107(66.0)	55(34.0)
	Stage III	48(11.3)	18(37.5)	30(62.5)
	Stage IV	81(19.1)	19(23.5)	62(76.5)
Metastasis	No	149(35.1)	117(78.5)	32(21.5)
	Local extension	168(39.6)	114(67.9)	54(32.1)
	Metastatic	107(25.2)	17(15.9)	90(84.1)
Intensity of background pain	No	46(10.8)	39(84.8)	7(15.2)
	Mild	130(30.7)	86(66.2)	44(33.8)
	Moderate	127(30.0)	72(56.7)	55(43.3)
	Severe	121(28.5)	51(42.1)	70(57.9)
Analgesic used for background pain	Yes	259(61.1)	172(66.4)	87(33.6)
	No	165(38.9)	76(46.1)	89(53.9)
Background pain	Controlled	268(63.2)	200(74.6)	68(25.4)
	Uncontrolled	156(36.8)	48(30.8)	108(69.2)
Cancer types	Breast cancer	103 (24.3)	37 (35.9)	66 (64.1)
	Colorectal cancer	34 (8.0)	22 (64.7)	12 (35.3)
	Liver cancer	24 (5.7)	9 (37.5)	15 (62.5)
	Ovarian cancer	36 (8.5)	17 (47.2)	19 (52.2)
	Cervical cancer	51 (12.0)	15 (29.4)	36 (70.6)
	Lung cancer	58 (13.7)	38 (65.5)	20 (34.5)
	Pancreatic cancer	30 (7.1)	13 (43.3)	17 (56.7)
	Gastric cancer	29 (6.8)	11 (37.9)	18 (62.1)
	Esophageal cancer	31 (7.3)	7 (22.6)	24 (77.4)
	Others	28 (6.6)	7 (25.0)	21 (75.0)

### Prevalence of breakthrough cancer pain

We found that 176 patients had BTCP (41.5%, 95% CI = 36.8 – 46.2). The prevalence of BTCP among female patients was 59.1%, whereas it was 40.9% among male patients. Among those 176 patients who had BTCP, 136 (77.3%) used medication to relieve BTCP, while the remaining 40 (22.7%) subjects did not use any medication to relieve their pain. The 56 (41.2%) patients were taking Morphine to relieve BTCP, while the remaining 42 (30.9%) and 38 (27.9%) took NSAID and NSAID with other combinations respectively (**Table 2**). The prevalence of BTCP was relatively high in the oncology unit at FHCSH (**Table 3**).

**Table 2:** Characteristics of breakthrough cancer pain, N = 424.

Variables	Category	Frequency	Percentage
Analgesics used for breakthrough cancer pain	Morphine	56	13.2
	NSAIDS	42	9.9
	NSAIDS and other	38	9.0
	No analgesics	288	67.9
Types of BTCP	Somatic nociceptive	57	13.4
	Visceral nociceptive	65	15.3
	Neuropathic	23	5.4
	Mixed	7	1.7
	No pain	272	64.2
Common pain localization	Abdominal	54	12.7
	Thoracic	35	8.3
	Extremities	19	4.5
Common triggers of breakthrough pain	Walking and Coughing	22	5.2
	Movement in bed	17	4.0
	Eating	15	3.5
	Coughing	14	3.3
Relieve from breakthrough pain	Sleeping and use of breakthrough pain medication	50	11.8
	Use of breakthrough pain medication	19	4.5
	Use of scheduled pain medication	19	4.5
	Touching/rubbing/squeezing painful area	13	3.1

BTCP, Breakthrough Cancer Pain; NSAIDS, Non-steroidal anti-inflammatory drugs.

**Table 3:** Prevalence of breakthrough cancer pain among oncology units, (N = 424).

Oncology units	Breakthrough cancer pain	
	Yes n (%)	No n (%)
UOGCSH	56 (41.8)	78 (58.2)
FHCSH	54 (48.2)	58 (51.8)
DCSH	66 (37.1)	112 (62.9)

UoGCSH, University of Gondar Comprehensive Specialized Hospital; FHCSH, Felege-Hiwot Comprehensive Specialized Hospital; DCSH, Dessie Comprehensive Specialized Hospital.

### Factors associated with breakthrough cancer pain

In the bivariate logistic regression analysis, cancer types, stage of cancer, metastasis, the intensity of background pain, the analgesic used for background pain, and uncontrolled background pain were found to be significantly associated with BTCP (P < 0.02) and fitted for the final analysis model. The multivariate logistic regression analysis showed that cancer types (colorectal and lung cancer), metastasis, intensity of background pain, the analgesic drugs used for background pain, and uncontrolled background pain were statistically associated with BTCP (P < 0.05) (**Table 4**).

**Table 4:** Bivariable and multivariable logistic regression analysis: factors associated with breakthrough cancer pain, N = 424.

Variables	Categories	Breakthrough cancer pain		Odds ratio		P-value
		Yes n (%)	No n (%)	COR (95% CI)	AOR (95% CI)	
Cancer types	Breast cancer	37 (35.9)	66 (64.1)	1.7 (0.7 – 4.3)	2.1 (0.6 – 7.2)	0.257
	Colorectal cancer	22 (64.7)	12 (35.3)	5.5 (1.8 – 16.7)	7.7 (1.8 – 32.3)	**0.005**
	Liver cancer	9 (37.5)	15 (62.5)	1.8 (0.6 – 5.9)	1.4 (0.3 – 7.0)	0.67
	Ovarian cancer	17 (47.2)	19 (52.2)	2.7 (0.9 – 7.9)	2.3 (0.5 – 9.5)	0.266
	Cervical cancer	15 (29.4)	36 (70.6)	1.3 (0.4 – 3.6)	1.2 (0.3 – 5.0)	0.773
	Lung cancer	38 (65.5)	20 (34.5)	5.7 (2.1 – 15.7)	6.9 (1.9 – 26.0)	**0.004**
	Pancreatic cancer	13 (43.3)	17 (56.7)	2.3 (0.8 – 7.0)	1.6 (0.3 – 7.5)	0.554
	Gastric cancer	11 (37.9)	18 (62.1)	1.8 (0.6 – 5.7)	1.7 (0.4 – 7.4)	0.472
	Esophageal cancer	7 (22.6)	24 (77.4)	0.9 (0.3 – 2.9)	2.5 (0.6 – 10.9)	0.23
	Others cancer	7 (25.0)	21 (75.0)	1	1	1
Stage of cancer	Stage I	29 (21.8)	104 (78.2)	1	1	1
	Stage II	55 (34.0)	107 (66.0)	1.8 (1.1 – 3.1)	1.3 (0.3 – 5.0)	0.728
	Stage III	30 (62.5)	18 (37.5)	5.9 (2.9 – 12.2)	0.9 (0.3 – 3.4)	0.975
	Stage IV	62 (76.5)	19 (23.5)	11.7 (6.1 – 22.6)	1.54 (0.5 – 4.7)	0.452
Metastasis	No	32 (21.5)	117 (78.5)	1	1	1
	Local extensión	54 (32.1)	114 (67.9)	1.7 (1.0 – 2.9)	0.7 (0.2 – 2.6)	0.564
	Metástasis	90 (84.1)	17 (15.9)	19.4 (10.1 – 37.1)	9.3 (3.0 – 29.1)	**< 0.001**
Intensity of background pain	No	7 (15.2)	39 (84.8)	1	1	1
	Mild	44 (33.8)	86 (66.2)	2.9 (1.2 – 6.9)	7.5 (2.5 – 22.6)	**< 0.001**
	Moderate	55 (43.3)	72 (56.7)	4.3 (1.8 – 10.2)	7.0 (2.2 – 23.1)	**0.001**
	Severe	70 (57.9)	51 (42.1)	7.7 (3.2 – 18.5)	7.1 (2.2 – 22.8)	**0.001**
Analgesics for background pain	Yes	87 (33.6)	172 (66.4)	1	1	1
	No	89 (53.9)	76 (46.1)	2.3 (1.6 – 3.5)	5.1 (2.8 – 9.3)	**< 0.001**
Background pain	Controlled	68 (25.4)	200 (74.6)	1	1	1
	Uncontrolled	108 (69.2)	48 (30.8)	6.6 (4.3 – 10.3)	3.3 (1.8 – 6.1)	**< 0.001**

COR, Crude Odds Ratio; AOR, Adjusted Odds Ratio; CI, Confidence Interval.

Bold = statistically significant in the multivariate logistic regression analysis.

Patients with colorectal and lung cancer had seven times higher odds of having BTCP **[AOR = 7.7, 95% CI = 1.8 – 32.3, P = 0.005]** and **[AOR = 6.9, 95% CI = 1.9 – 26.0, P = 0.004]** respectively. The presence of metastasis increased the odds of having BTCP by nine times **[AOR = 9.3, 95% CI = 3.0 – 29.1, P < 0.001]**. Patients who were not taking analgesics for background pain were found to have BTCP in fivefold more cases **[AOR = 5.1, 95% CI = 2.8 – 9.3, P < 0.001]**. Having background pain of any intensity increased the odds of having BTCP by over seven times [P ≤ 0.001]. Similarly, having uncontrolled pain was found to predispose patients to BTCP by triple fold **[AOR = 3.3, 95% CI = 1.8 – 6.1, P < 0.004]** (**Table 4**).

## Discussion

Breakthrough pain is frequent in cancer patients and is often characterized by a rapid onset, short duration, and severe intensity (5, 40). In this study, the prevalence of breakthrough cancer pain was 41.5% and it was consistent with previous studies that reported 39.3% (41), 41% (42), and 40.3% (43). However, it was considerably higher compared to other studies that reported 21% (44) and 29% (45). This discrepancy could be explained by the differences in the study settings. Access to quality cancer pain treatment and palliative care can be restricted in low-income countries (46). Furthermore, conflict affected areas are expected to suffer from disrupted cancer care due to damaged infrastructures, shattered supply chains, financial burdens, and evacuation of the health workforce. Similar to Northern Ethiopia, this finding is evidenced by recent studies in Ukraine and the Middle East (47, 48). In contrast, the prevalence of BTCP in the current study was relatively lower than that of other studies at 64.8% (49), 69.8% (22), 73%, and 79% (50).

Among cancer types, colorectal and lung cancer were found to be significantly associated with BTCP. This finding was supported by previous studies and justified by worsening pain and discomfort during coughing, laughing, deep breathing, straining, and defecation (28, 51, 52).

Patients who had metastasis were highly likely to have BTCP and previous studies have supported this finding (10, 49). This condition might be explained by metastatic cancer affecting multiple organs and systems. The size and rate of growth are two important factors that determine pain due to metastatic cancer. Patients could be affected by BTCP and its negative consequences, such as reduced daily activity, worsened quality of life, and dissatisfaction. Patients with metastasis are more likely to receive chemotherapy and radiation therapy, which can result in pain flare-ups (23).

Background pain of any intensity (mild, moderate, or severe) was associated with BTCP. It increased the likelihood of having BTCP by seven times. The association between background pain and BTCP has been reported in multiple previous studies (23, 27, 53, 54). Cancer patients frequently experience pain, which has a favorable correlation with breakthrough pain. Untreated background pain causes a release of an excitatory neurotransmitter such as glutamate, which facilitates the development of BTCP (55).

If patients did not take analgesics to treat background pain, they were highly likely to develop BTCP. A large international survey in twenty-four countries showed that BTCP predominantly occurred among patients who did not take analgesics to treat background pain compared to those who did (49). Multiple previous studies also supported this finding (22, 56–58). Tumor compression against bones, nerves, and other tissues causes the release of chemicals that initiate and mediate pain. The administration of analgesics for cancer patients helps to heal the damaged tissue and prevents the release of those chemicals (5, 23). Patients who had uncontrolled background pain were more likely to complain of BTCP (59–61).

This multi-center study was conducted in a research-scarce part of the world on breakthrough cancer pain. However, it did not establish temporal and causal relationships between factors and breakthrough cancer pain due to the cross-sectional nature of the study design. Statistical analysis was performed with logistic regression which could overestimate the odds ratio as the prevalence of BTCP was higher than 10%. In addition, we measured only spontaneous breakthrough pain but not incident pain during certain triggering activities such as motion. Furthermore, the inclusion of heterogeneous patients with different typologies of cancer might have limited the generalizability of the findings.

## Conclusion

There is a high prevalence of breakthrough cancer pain in oncology units in Northern Ethiopia. Colorectal and lung cancer, metastasis, intensity of background pain, not using analgesics for background pain, and uncontrolled background pain were found to be significantly associated with breakthrough cancer pain. It is advised to regularly assess cancer patients for breakthrough cancer pain and provide adequate pain management.

## Data availability statement

Data and materials used in this study are available and can be presented by the corresponding author upon reasonable request.

## Ethics statement

Ethical approval was obtained from the Ethical Review Committee of the School of Medicine, University of Gondar (Reference number: SOM/1404/2022). Permission to conduct data collection was obtained from each hospital. Written informed consent was obtained from each study participant after a detailed explanation of the study. All methods were performed in accordance with the relevant research guidelines and regulations.

## Author contributions

YT conceptualized the study and objectives and developed the proposal. YB, YN, and WM criticized the proposal. All authors participated in data management and statistical analyses. YB and YT led the manuscript preparation. All authors contributed to the article and approved the submitted version.
